# A collaborative study of the impact of N-nitrosamines presence and ARB recall on ARB utilization – results from IQVIA™ Disease Analyzer Germany

**DOI:** 10.1007/s00228-022-03439-3

**Published:** 2023-04-24

**Authors:** Karin Hedenmalm, Chantal Quinten, Xavier Kurz, Marie Bradley, Hana Lee, Efe Eworuke

**Affiliations:** 1 Data Analytics Workstream, Data Analytics and Methods Task Force, European Medicines Agency, Amsterdam, Netherlands; 2 Department of Laboratory Medicine, Karolinska Institutet, Solna, Stockholm Sweden; 3 Center for Drug Evaluation and Research, Food and Drug Administration, Silver Spring, USA

**Keywords:** Drug utilization, Angiotensin receptor blockers, Drug recalls, N-nitrosamines

## Abstract

**Purpose:**

Regulators are increasingly concerned with  the impact of recalls on drug adherence. In 2018, N-nitrosamines impurities were detected in valsartan containing medical products. Concerned products were immediately recalled in July 2018 by regulatory agencies internationally. In Germany, recalls were issued for valsartan, losartan and irbesartan from July 2018 to March 2019. This study examined angiotensin II receptor blocker (ARB) utilization trends and switching patterns in Germany before and after July 2018.

**Methods:**

Patients prescribed ARBs from January 2014 to June 2020 in general practices in Germany were included in a collaborative framework common protocol drug utilization study led by the US Food and Drug Administration. Trends in monthly and quarterly proportions of total ARB prescribing were analysed for individual ARBs using descriptive statistics and interrupted time series analysis. The rate of switching to an alternative ARB was analysed before and after the recalls.

**Results:**

The proportion of valsartan prescriptions immediately decreased from 35.9 to 17.8% following the first recalls in July 2018, mirrored by an increased proportion for candesartan. Increased switching from valsartan to candesartan was observed. No increased switching was observed after losartan recalls, whereas for irbesartan, increased switching was observed 6–12 months after the last recall. Increased switching from ARBs to angiotensin-converting enzyme (ACE) inhibitors or ARB treatment discontinuations were not observed.

**Conclusion:**

This study showed that patients were able to continue ARB treatment despite the July 2018–March 2019 recalls, although many patients needed to switch to an alternative ARB. The duration of the impact of ARB recalls appeared to be limited.

**Supplementary Information:**

The online version contains supplementary material available at 10.1007/s00228-022-03439-3.

## Introduction

Valsartan is an angiotensin II receptor blocker (ARB) indicated for treatment of hypertension, recent heart attack and heart failure. On 5 July 2018, the European Medicines Agency (EMA) issued a press release to communicate the recall of some medicines containing valsartan as active pharmaceutical ingredient (API) manufactured by Zheijiang Huahai Pharmaceutical in China. The nitrosamine N-nitrosodimethylamine (NDMA) impurity was detected in the API. The synthesis of a tetrazole ring structure was identified as a source of N-nitrosamines (hereafter referred to as nitrosamine) impurities in some API manufacturing processes for certain ARBs [1], raising questions about long-term safety as a consequence of increased exposure to potential carcinogens [2, 3]. Currently, multiple risk factors have been identified for presence of nitrosamines in APIs [4] which are regularly re-evaluated as additional knowledge and scientific data becomes available. A possible link between nitrosamines and human cancer was identified already during the early 1980’s for tobacco-specific nitrosamines formed from nicotine in tobacco products [5]. NDMA and nitrosamine N-nitrosodiethylamine (NDEA) are classified by the International Agency for Research on Cancer (IARC) as probable carcinogens to humans [6]. A review of possible impurities by NDMA and NDEA was conducted for the entire class of ARBs, which resulted in further recalls of other ARBs. Patients treated with ARBs were informed to continue their treatment unless told to stop by their health care professional. Actions were also taken by the United States Food and Drug Administration (US FDA) and other regulatory agencies [7, 8].

In parallel with the EMA press release, the German Federal Institute for Drugs and Medical Devices announced the initiation of ARB recalls in Germany [9]. Recalls were issued for valsartan, irbesartan and losartan [10] between July 2018 and March 2019.

Regulatory agencies are increasingly interested in the impact of recalls on drug adherence and use. Therefore, this study investigated the impact of recalls of ARB-containing medicines between 2018 and 2019 in Germany on prescribing of individual ARBs in relation to total ARB prescribing and on switching from one ARB to an alternative ARB or an angiotensin-converting enzyme (ACE) inhibitor. This study was undertaken by the EMA using electronic health records from Germany available in-house as part of a collaborative protocol initiated by the FDA and adapted to the data available at EMA.

## Methods

### Study design, setting and participants

A drug utilization study was conducted between January 2014 and June 2020 using descriptive statistics, supplemented by an interrupted time series (ITS) analysis to study changes in prescribing trends for individual ARBs before and after the first ARB recalls in July 2018. All patients with at least one ARB prescription during the study period were included in the study. Prescribing of individual ARBs over time was studied as the monthly proportion of all ARB use and as the quarterly proportion of incident ARB use, respectively.

Patients with at least two ARB prescriptions on separate dates during the study period with a minimum look-back period of 365 days prior to the first ARB prescription (referred to as the first ARB treatment episode) during the study period were followed up until they switched to another ARB or an ACE inhibitor, discontinued the ARB treatment, or were lost to follow-up (end of data). For this purpose, the first ARB prescription date during the study period was the index date for the patient. Patients prescribed more than one ARB (e.g. both valsartan and candesartan) on the index date (*n* = 246) were excluded. For each individual ARB, the proportion of switches was considered in relation to all patients with ARB use during the quarter. The pattern of ARBs prescribed after the switch (i.e. the ARB that the patient switched to) was also assessed. Finally, the rate of switching to an alternative ARB during person-time of follow-up was analysed before and after the ARB recalls.

### Data source

The study was based on prescribing data from general practitioner (GP) practices in the IQVIA™ Disease Analyzer database in Germany (DA Germany).

The study used DA Germany version June 2020. DA Germany collects computerised information from specialised and GP practices throughout Germany since 1992. Around 3% of GP practices from the different regions in Germany are included in the database. Data from DA Germany have been shown to be reasonably representative of German healthcare statistics for demographics and certain diseases [11, 12].

DA Germany contains information about prescriptions issued by physicians, whereas dispensing information from pharmacies is not available. Prescribed medicines in DA Germany are coded using EphMRA ATC codes and generic substance names for the active ingredients.

### Exposure variables

ARBs were identified using EphMRA ATC codes C09C (angiotensin II antagonists, plain) and C09D (angiotensin II antagonists, combinations). ACE inhibitors were identified using EphMRA ATC codes C09A (ACE inhibitors, plain) and C09B (ACE inhibitors, combinations). Information on the prescribed ARB was used. The pharmacy could dispense an alternate product, with the same ARB, strength and formulation. ARBs prescribed in DA Germany included azilsartan, candesartan, eprosartan, irbesartan, losartan, olmesartan, telmisartan and valsartan.

### Measurements

The measures of interest were individual ARB use over time and switching from the index ARB to an alternative ARB or an ACE inhibitor. Switching from the index ARB to an alternative ARB or an ACE inhibitor was studied during the first ARB treatment episode.

#### Individual ARB use over time: monthly proportion of total ARB use

The total number of ARB prescriptions was estimated each month during the study period. The proportion of all ARB prescriptions was calculated for each individual ARB.

#### Individual ARB use over time: quarterly proportion of incident ARB use

Incident ARB use was defined as the first ARB prescription during the study period in a patient with no previous ARB prescription during the preceding 365 days. The total number of patients with incident ARB use was estimated quarterly during the study period. The proportion of all patients with incident ARB use was calculated for each individual ARB.

#### Switching from the index ARB to an alternative ARB or an ACE inhibitor

A patient was considered to have switched to another ARB if another ARB was prescribed at any time during the first ARB treatment episode. Switching to an ACE inhibitor was only considered in patients that did not switch to another ARB and stopped treatment with the index ARB, to avoid classifying add-on treatment as a switch. The length of the first ARB treatment episode until switching to an alternative ARB, an ACE inhibitor, or treatment discontinuation was estimated for each patient by dividing the total number of tablets by the prescribed daily dose and allowing a maximum gap of 183 days (6 months) between calculated prescription dates. For prescriptions with no recorded daily dose, the median dose for the specific product was used to impute the daily dose or the median dose for all products with the same composition from the same manufacturer, where available, or the defined daily dose [13]. The end date of the treatment episode was the earliest of the end of patient observability (i.e. the last visit of the patient to the practice), database end, 183 days after the calculated end date of the last prescription during the treatment episode, the date of switching to an alternative ARB or the date of switching to an ACE inhibitor. Sensitivity analyses were also carried out, allowing a maximum gap of 90 days or 270 days instead of 183 days.

It was assumed to take up to 6 months after the recall for the switching to occur if patients treated with contaminated ARBs did not switch until the time of the next prescription, as 6 months was the maximum allowed gap between prescription dates during the treatment episode. Hence, a post-recall time period between 11 July 2018 and 20 June 2019 was considered for valsartan (last recall 20 December 2018) [10], 16 July 2018 to 19 August 2019 for irbesartan (last recall 19 February 2019) [10] and 28 December 2018 to 4 September 2019 for losartan (last recall 4 March 2019) [10]. In a sensitivity analysis, the post-recall time period was extended by a further 6 months.

### Statistical methods

Prescribing patterns of individual ARBs, as monthly proportion of total ARB use and as quarterly proportion of incident ARB use, were analysed descriptively and by ITS. [14] ITS examines changes in trends before and after an intervention (i.e. the recall) as well as changes in values right before and after the time of the intervention (i.e. the level change). ITS analysis was performed for ARBs that had a minimum proportion of 2% of all ARB use (i.e. candesartan, irbesartan, losartan, olmesartan, telmisartan and valsartan). All analyses were conducted using SAS Enterprise Guide version 7.15.

We also conducted comparative ITS analyses of the difference in ARB proportions [14], comparing valsartan (reference) to candesartan, irbesartan, losartan, olmesartan and telmisartan. July 2018 (the time of the first ARB recall) was selected as an intervention time point for all ITS analyses. A sensitivity analysis was also conducted, using the same number of pre- and post-intervention time points to maximize power (i.e. data from September 2016 for the monthly analysis and data from the first quarter of 2017 for the quarterly analysis) as well as to reduce the influence of historic changes.

Switching was analysed descriptively as a proportion of quarterly ARB treatment episodes leading to a switch during the entire study period. In addition, the rate of switching, defined as the number of ARB treatment episodes leading to a switch per person-year of follow-up, was analysed before and after the ARB recalls, and rates were compared using the *z*-score test [15–17]. The analyses in the study were performed by the authors.

## Results

### Total ARB prescribing

A total of 5,740,602 ARB prescriptions were identified in 552,512 patients in GP practices during the study period of which 257,036 patients (46.5%) were incident users.

### Individual ARB use over time as proportion of all ARB use

**Fig. 1:**
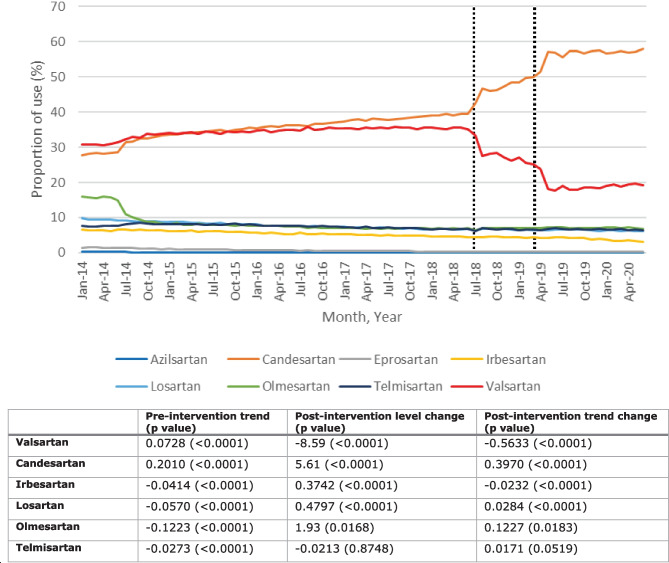
Upper part: monthly proportion of individual angiotensin II receptor blockers (ARBs) out of all ARB prescriptions between January 2014 and June 2020 in IQVIA™ Disease Analyzer Germany**.** Lower part: results of interrupted time series analysis of change in monthly percentage for individual ARBs after July 2018. The dotted lines show the first and last recall dates. Only ARBs with a minimum proportion of 2% of all ARB prescriptions were included in the analysics

The monthly proportion of individual ARB use over time is shown in Fig. 1, alongside the results of ITS analyses.

Valsartan and candesartan were the most commonly prescribed ARBs. Prior to the first ARB recall, the proportion for valsartan varied from 30.6 to 35.9%, and thereafter it varied from 17.8 to 28.4%, with the lowest proportion observed in June 2019. For candesartan, the proportion varied from 27.6 to 42.6% prior to the first ARB recall, and thereafter it varied from 46.0 to 58.0%, with the highest proportion observed in June 2020. For the other ARBs, proportions were generally below 10%.

Prior to the first ARB recall, trends were increasing for valsartan and candesartan and decreasing for other ARBs. Both valsartan and irbesartan had significant decreases in trend after the first ARB recall, but only valsartan also had a significant decrease in level. The decreases in level and trend for valsartan were significant compared to the other ARBs in the comparative ITS analysis for valsartan vs. each of the other ARBs (Supplementary Information, Table S1).

Results of the sensitivity analysis that used the same number of pre- and post-intervention time points (Supplementary Information, Fig. S1 and Table S2) were similar to the main ITS analysis, but the pre-ARB recall trend for valsartan was no longer significant.

### Individual ARB use over time as proportion of incident ARB use

**Fig. 2:**
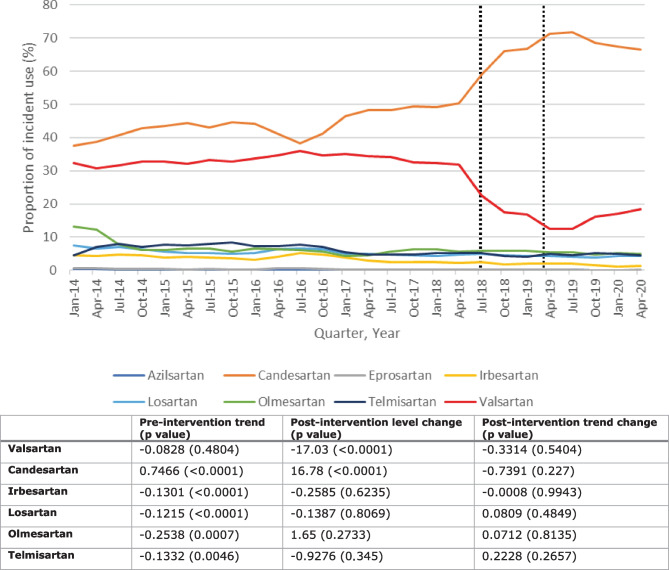
Upper part: quarterly proportion of individual angiotensin II receptor blocker (ARB) users out of all incident ARB users between January 2014 and June 2020 in IQVIA™ Disease Analyzer Germany. Lower part: results of interrupted time series analysis of change in quarterly percentage for individual ARBs after July 2018. The dotted lines show the first and last recall dates. Only ARBs with a minimum proportion of 2% of all ARB prescriptions were included in the analysis

The quarterly proportion of individual incident ARB use over time is shown in Fig. 2 with the results of ITS analyses shown below the graph.

The decrease in the proportion of incident valsartan use (from 22.6–35.9 to 12.4–18.4%) was more pronounced compared to all valsartan use.

No significant changes in trend were observed, and the pre-ARB recall trend for valsartan was not significant. A substantial decrease in the level of valsartan was observed compared to the other ARBs (Supplementary Information, Table S3).

Results of the sensitivity analysis that used the same number of pre- and post-intervention time points (Supplementary Information, Fig. S2 and Table S4) were similar to the main ITS analysis, but a decreasing pre-ARB recall trend was noted for valsartan.

### Duration of ARB treatment

A total of 329,472 patients (204,244 incident users) had an ARB treatment episode during the study period. Patients received a median of 9 prescriptions of the index ARB prior to any switch. The median duration of the ARB treatment episode during the study period was 98 days and was the same for all ARBs. Using gaps of 183 days in the main analysis, the average quarterly proportion of discontinuations during the study period for all ARBs was 1.90%. The proportion of discontinuations in the sensitivity analyses was higher for shorter allowed gaps (4.19%) and lower for longer allowed gaps (1.28%) (Supplementary Information, Fig. S3).

### Switching to an alternative ARB

**Fig. 3:**
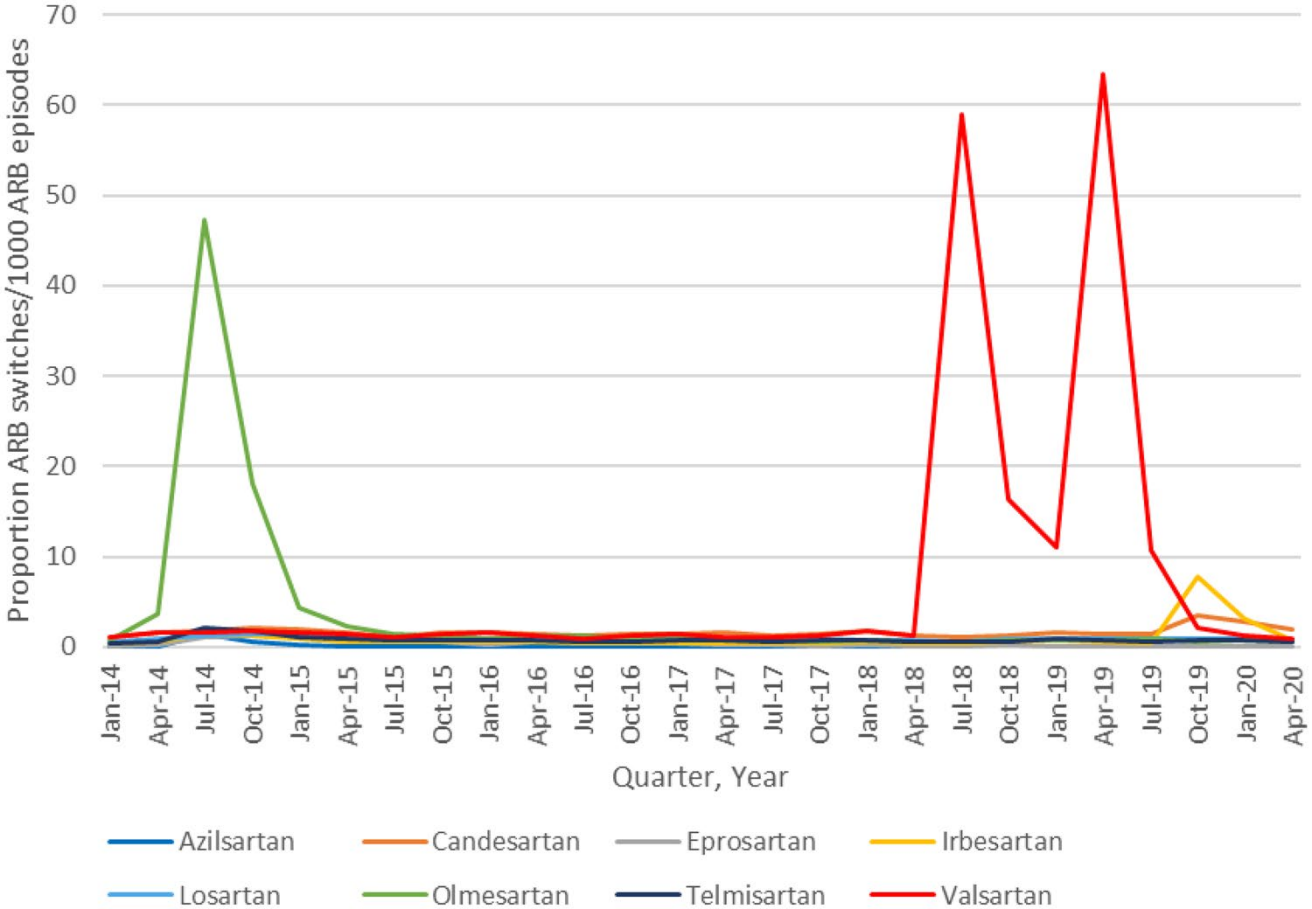
Quarterly switching from an individual index angiotensin II receptor blocker (ARB) to an alternative ARB as proportion of total quarterly ARB episodes between January 2014 and June 2020 in IQVIA™ Disease Analyzer Germany

Quarterly switching from an individual index ARB to an alternative ARB as a proportion of total ARB episodes over time is shown in Fig. 3.

Using a maximum gap of 90 or 270 days instead of 183 days between calculated treatment days in the sensitivity analyses did not change the results (data not shown). For ARBs affected by recalls (valsartan, irbesartan and losartan), an analysis of the rate of switching is shown in Table 1.

**Table 1:** Rate of switching from an index angiotensin II receptor blocker (ARB) affected by recalls to an alternative ARB, before and after the initiation of recalls

**Index ARB**^**a**^	**Pre-recall switching rate**^**b**^	**Post-recall switching rate**^**b**^	**P value for difference between periods**
Irbesartan^c^	0.043 (1,572/36,942)	0.042 (383/9,013)	0.98
Losartan^d^	0.048 (2,721/57,241)	0.050 (372/7,412)	0.33
Valsartan^e^	0.018 (3,845/211,033)	0.592 (23,805/40,243)	< 0.0001

^a^The ARB prescribed during the treatment episode

^b^Switching rate was the number of episodes leading to a switch per person-year of follow-up (values in parenthesis). Follow-up time was rounded to nearest integer

^c^Pre-recall time period: 1 January 2014 to 15 July 2018. Post-recall time period: 16 July 2018–19 August 2019. In a sensitivity analysis, the post-recall time period was extended to 19 February 2020: 1,853 switches were observed during 12,214 years of follow-up (switching rate 0.152; *p* value < 0.0001 for the difference between periods)

^d^Pre-recall time period: 1 January 2014 to 27 December 2018. Post-recall time period: 28 December 2018–4 September 2019. In a sensitivity analysis, the post-recall time period was extended to 4 March 2020: 620 switches were observed during 12,477 years of follow-up (switching rate 0.050; *p* value 0.32 for the difference between periods)

^e^Pre-recall time period: 1 January 2014 to 10 July 2018. Post-recall time period: 11 July 2018–20 June 2019. In a sensitivity analysis, the post-recall time period was extended to 20 December 2019: 26,203 switches were observed during 54,077 years of follow-up (switching rate 0.485; *p* value < 0.0001 for the difference between periods)

A significant increase in switching after vs before the recalls was only observed for valsartan (around 30-fold). However, in the sensitivity analysis where the time period of follow-up was increased to 12 instead of 6 months after the last recall, a significant increase in switching was also observed for irbesartan (around threefold).

### Pattern of ARB switching

**Fig. 4:**
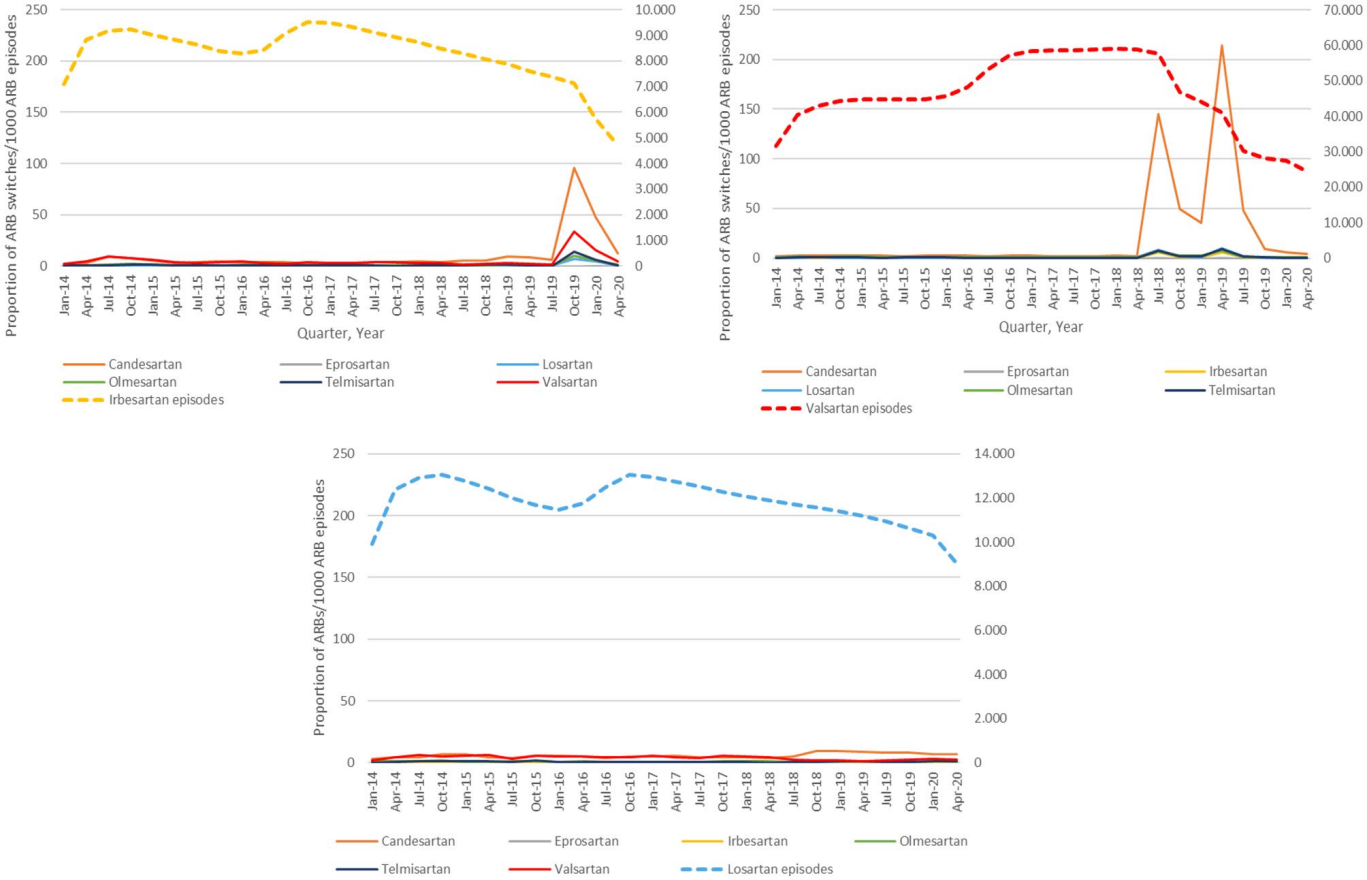
Quarterly switching patterns inusers of individual angiotensin II receptor blockers (ARBs) that were subject to recalls between 2018 and 2019. Switches to azilsartan are not shown because fewer than 10 patients had a switch to azilsartan. For irbesartan, recalls were issued between 16 July 2018 and 19 February 2019. For losartan, recalls were issued between 28 December 2018 and 4 March 2019. For valsartan, recalls were issued between 16 July 2018 and 20 December 2018

The pattern of ARB switching in users of index ARBs subject to recalls is shown in Fig. 4. Most of the patients that switched during the ARB recall time period switched to candesartan.

### Switching to ACE inhibitor and ARB treatment discontinuation

Switching from an ARB to an ACE inhibitor remained low throughout the study period (Supplementary Information, Fig. S4). There was no evidence of an increase in ARB treatment discontinuations after July 2018 (Supplementary Information, Fig. S3).

## Discussion

Our study showed a rapid and pronounced decline in the proportion of valsartan use following the first ARB recalls in July 2018 in Germany. The impact was, however, of limited duration, which could be expected when recalled products containing nitrosamines above acceptable levels were, thanks to testing activities at the company level, increasingly replaced by unrecalled products with the same ARB over time that fulfil the requirements for acceptable nitrosamine levels. After the last valsartan recall in December 2018, the proportion of valsartan use stabilized in the summer of 2019 with no increased switching after the third quarter of 2019. Most switching was to candesartan, which was the most frequently prescribed ARB after the recalls. Changes in the proportion of candesartan use were almost a mirror image of the changes for valsartan. The rate of switching in valsartan users increased around 30-fold. The recalls had a stronger impact on incident valsartan use (65.5%; from 35.9 to 12.4%) vs. all valsartan use (50.5%; from 35.9 to 17.8%). These rapid profound changes resulted from a relatively high proportion of use of valsartan at the start of the study period in combination with a significant market share for the contaminated products that were recalled.

All ITS analyses supported a significant decrease in valsartan utilization compared to other ARBs, although in the quarterly analysis, due to a decreasing number of time points and the fact that the first ARB recall occurred at the beginning of a quarter, a significant decrease in the trend was not observed. As it is preferable to have an equal distribution of data points before and after the intervention to maximize power [18], we also conducted a sensitivity analysis that used the same number of data points before and after the first ARB recall, i.e. data from September 2016 for the monthly analysis and January 2017 for the quarterly analysis. It can be noted that an increasing proportion of use of valsartan at the beginning of the study period, from 30.71% in January 2014 to 35.85% in August 2016, was replaced by a more stable proportion of use between September 2016 and June 2018 (from 34.91 to 35.87%) and a decrease to 33.45% during the first month of the ARB recalls in July 2018. In the sensitivity analysis using monthly data, a negative post-ARB recall trend was still observed for valsartan whereas in the quarterly data the post-ARB recall trend for valsartan was increasing compared to a decreasing pre-ARB recall trend. Despite these valsartan prescribing changes, there was no evidence of an increase in ARB discontinuations (Fig. S3).

Smaller changes were observed for the other ARBs. The recalls for losartan had no impact on switching from losartan to alternative ARBs, and for irbesartan there was no evidence of switching up to 6 months after the last recall. Between 6 and 12 months after the last irbesartan recall, however, a threefold increase in switching to alternative ARBs was observed. This finding might indicate fewer patients seeking to immediately replace a recalled drug for example due to a low initial market share for recalled products followed by a later occurring shortage, possibly due to limited replacement of recalled products by unrecalled products, or the finding might be unrelated to the presence of nitrosamine impurities. For olmesartan, a decrease in the proportion of olmesartan use and an increased switching from olmesartan to alternative ARBs was observed in 2014 that coincided with publications of the risk of sprue-like enteropathy for olmesartan [19, 20].

 Overall, a risk of cancer due to the presence of nitrosamine impurities in medicines is regarded as low [25, 27–31] but cannot be excluded [32, 33]. Following the experience with ARBs, the EMA has developed a number of policies and recommendations to companies, supported on evolving scientific data, to mitigate the risk of nitrosamine impurities in all medicines in order to protect public health and ensure patients access to medicines [25].

Results from this study were largely consistent with results from a previous study in Germany that also looked at impurities-related changes in ARB use [34], although olmesartan made a greater contribution to total ARB prescribing in our study. Our study also looked at switching directly based on individual patient prescribing data whereas the previous study inferred switching based on dispensing patterns. Lastly, our study had a longer follow-up time, which allowed observation of the increased switching in irbesartan patients 6–12 months after the last recall of irbesartan. A similar finding in both studies was the staggered decrease in the use of valsartan, with a rapid decrease in the summer of 2018 followed by relative stabilisation and another rapid decrease in the spring of 2019. Also, both studies showed that there was no increased switching to ACE inhibitors, and an increase in ARB treatment discontinuations after July 2018 was also not observed, indicating that patients were able to remain on ARB treatment. Finally, the previous study was based only on statutory health-insured patients whereas our study also included patients from private insurers, which is more representative of the entire German population.

Strengths of this study include the use of patient-level data and international collaboration with FDA based on a common protocol. The main limitations include access to only a random sample of GPs (around 3%), and to prescribed rather than dispensed ARBs. We were therefore unable to confirm that patients filled or received the products in the prescription. The reasons for switching were also not captured, although the use of a control period prior to the recalls provides some reassurance that the ARB utilization changes were due to the recall notices.

In conclusion, the results of this study indicate that patients were able to continue to be prescribed ARB despite recalls for potential nitrosamine impurities, although many patients needed to be switched to an alternative ARB with most switching seen between July 2018 and September 2019. Regulatory actions including recalls are necessary to safeguard patients, but it is important to study their consequences for prescribers and patients.

## Supplementary Information

Below is the link to the electronic supplementary material.

Supplementary file1 (DOCX 128 KB)

## Data Availability

Data in the study is based on version June 2020 of the IQVIA™ Disease Analyzer Germany database (IQVIA Copyright 2021. All rights reserved). Further information about the data and conditions for access are available at the [https://www.iqvia.com/-/media/iqvia/pdfs/library/fact-sheets/iqvia-health-data-catalog-fact-sheet.pdf?_=1666079784461] at iqvia.com.

## Declarations

### Disclaimer

The views expressed in this article are the personal views of the author(s) and may not be understood or quoted as being made on behalf of or reflecting the position of the regulatory agency/agencies or organisations with which the author(s) is/are employed/affiliated.
