# Supplementary material for: A collaborative study of the impact of N-nitrosamines presence and ARB recall on ARB utilization – results from IQVIA™ Disease Analyzer Germany
Source: Eur J Clin Pharmacol. 2023 Apr 24;79(6):849–58. doi: 10.1007/s00228-022-03439-3 (PMC10229727; doi:10.1007/s00228-022-03439-3)
Supplement: Supplementary file 1 — Supplementary file1 (DOCX 128 KB) [file 228_2022_3439_MOESM1_ESM.docx]

**Supplementary information**

**Article title:** A collaborative study of the impact of Nitrosamine Contamination and ARB Recall on ARB Utilization–

Results from IQVIA^TM^ Disease Analyzer Germany.

**Journal name:** European Journal of Clinical Pharmacology.

**Authors:** Karin Hedenmalm, Chantal Quinten, Xavier Kurz, Marie Bradley, Hana Lee, Efe Eworuke.

**Corresponding author:** Karin Hedenmalm.

**Email address of corresponding author:** [karin.hedenmalm@ema.europa.eu](mailto:karin.hedenmalm@ema.europa.eu)

Supplementary information has been provided for results of sensitivity analyses (Fig. S1-S4), for results of switching to an alternative angiotensin receptor blocker vs. switching to an angiotensin converting enzyme inhibitor (Fig. S4), and for results of comparative interrupted time series analyses (Tables S1-S4).

**Supplementary Figures:**

***Fig S 1:*** ***Upper part:*** *Monthly proportion of individual angiotensin II receptor blockers (ARBs) out of all ARB prescriptions between September 2016 and June 2020 in IQVIA^TM^ Disease Analyzer Germany.* ***Lower part:*** *Results of interrupted time series analysis of change in monthly percentage for individual ARBs after July 2018.*

The dotted lines show the first and last recall dates. Only ARBs with a minimum proportion of 2% of all ARB prescriptions were included in the analysis.

| 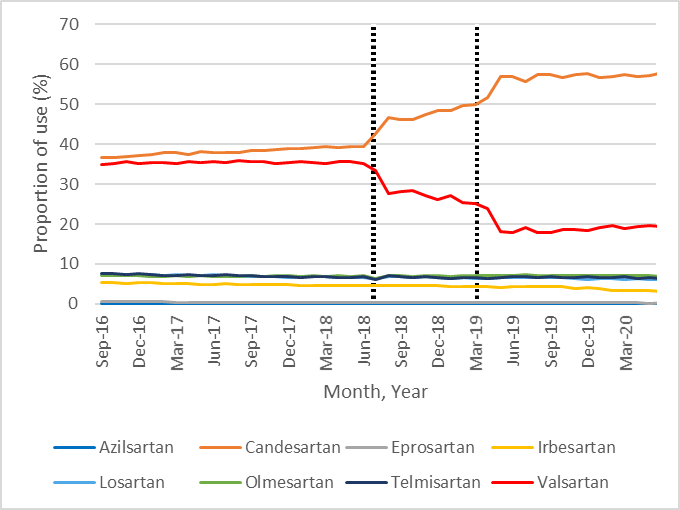 |
| --- |
| \|  \| Pre-intervention trend (p value) \| Post-intervention level change (p value) \| Post-intervention trend change (p value) \| \| --- \| --- \| --- \| --- \| \| Valsartan \| -0.0129 (0.811) \| -7.49 (<0.0001) \| -0.4777 (<0.0001) \| \| Candesartan \| 0.1649 (0.0017) \| 6.27 (<0.0001) \| 0.4331 (<0.0001) \| \| Irbesartan \| -0.0418 (<0.0001) \| 0.3863 (<0.0001) \| -0.0229 (0.0018) \| \| Losartan \| -0.0412 (<0.0001) \| 0.2829 (<0.0001) \| 0.0125 (0.0113) \| \| Olmesartan \| -0.111 (0.0052) \| 0.269 (0.0005) \| 0.0115 (0.0365) \| \| Telmisartan \| -0.0483 (<0.0001) \| -0.2809 (0,0024) \| 0.0381 (<0.0001) \| |

***Fig S 2:*** ***Upper part:*** *Quarterly proportion of individual angiotensin II receptor blocker (ARB) users out of all incident ARB users between January 2017 and June 2020 in IQVIA^TM^ Disease Analyzer Germany.* ***Lower part:*** *Results of interrupted time series analysis of change in quarterly percentage for individual ARBs after July 2018.*

The dotted lines show the first and last recall dates. Only ARBs with a minimum proportion of 2% of all ARB prescriptions were included in the analysis.

| 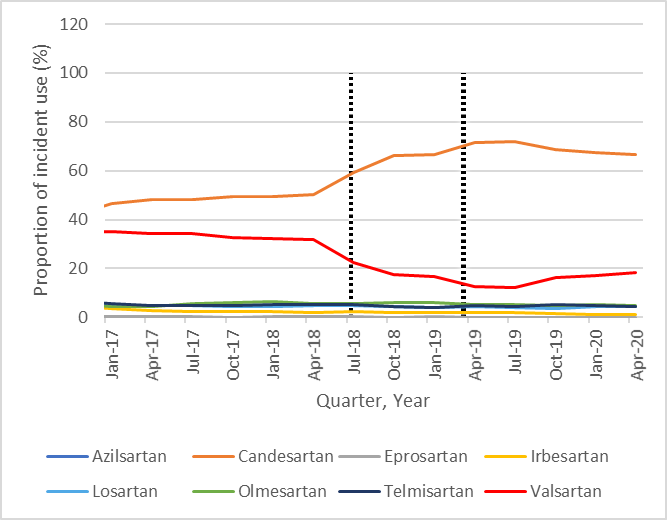 |
| --- |
| \|  \| Pre-intervention trend (p value) \| Post-intervention level change (p value) \| Post-intervention trend change (p value) \| \| --- \| --- \| --- \| --- \| \| Valsartan \| -1.57 (0.0114) \| -12.29 (0.0018) \| 1.82 (0.0297) \| \| Candesartan \| 0.154 (0.0111) \| 13.5 (0.0008) \| -1.53 (0.0535) \| \| Irbesartan \| -0.1934 (0.0041) \| 0.1517 (0.6248) \| 0.0641 (0.4068) \| \| Losartan \| -0.256 (0.6368) \| -0.302 (0.3413) \| 0.0151 (0.8437) \| \| Olmesartan \| 0.2528 (0.0149) \| -0.2161 (0.6714) \| -0.4354 (0.005) \| \| Telmisartan \| 0.0145 (0.8322) \| -0.7796 (0,0686) \| 0.0751 (0.443) \| |

**Fig S 3**: Quarterly proportion of treatment episodes with angiotensin II receptor blockers that resulted in discontinuation of treatment for different maximum gaps between calculated treatment days in IQVIA^TM^ Disease Analyzer Germany


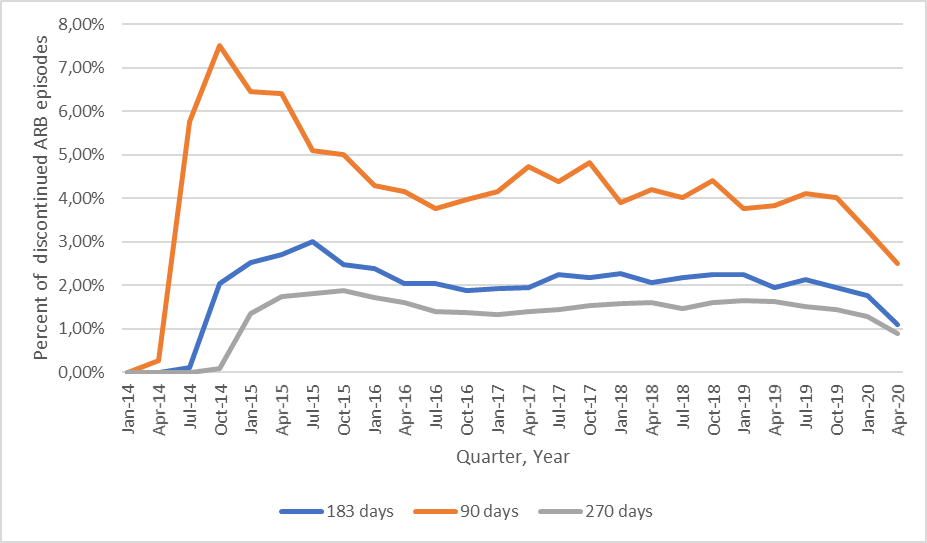


**Fig S 4:** Quarterly proportion of treatment episodes with angiotensin II receptor blockers that resulted in switching to an alternative ARB or an ACE inhibitor in IQVIA^TM^ Disease Analyzer Germany


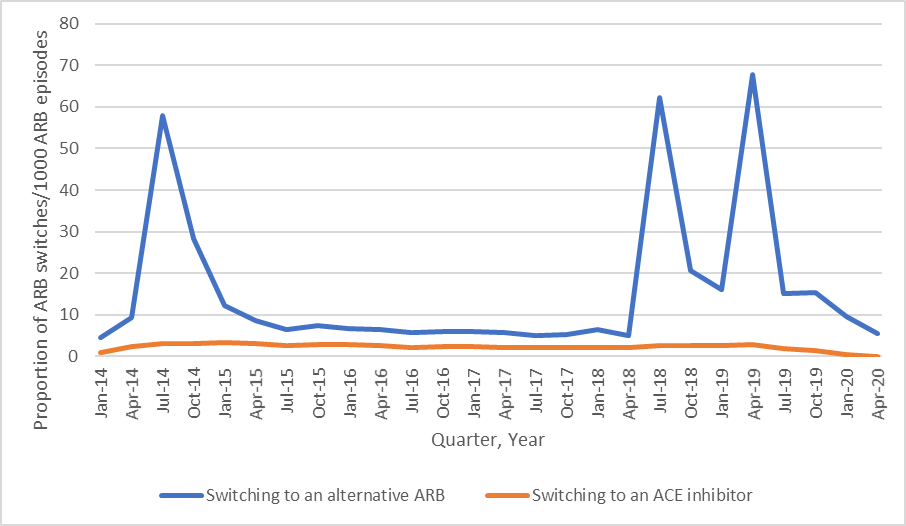


**Supplementary Tables**

Table S 1 Comparative interrupted time series analysis of data between January 2014 and June 2020: Change in monthly percentage of angiotensin II receptor blocker (ARB) prescriptions for valsartan vs other ARBs after July 2018 ^1^

| Comparator | Pre-intervention trend (p value) ^2^ | Post-intervention level change (p value) ^2^ | Post-intervention trend change (p value) ^2^ |
| --- | --- | --- | --- |
| Candesartan | -0.1283 (<0.0001) | -14.20 (<0.0001) | -0.9604 (<0.0001) |
| Irbesartan | 0.1142 (<0.0001) | -8.96 (<0.0001) | -0.5401 (<0.0001) |
| Losartan | 0.1298 (<0.0001) | -9.07 (<0.0001) | -0.5917 (<0.0001) |
| Olmesartan | 0.195 (<0.0001) | -10.52 (<0.0001) | -0.686 (<0.0001) |
| Telmisartan | 0.100 (<0.0001) | -8.57 (<0.0001) | -0.5805 (<0.0001) |

^1^ Only ARBs with a minimum proportion of 2% of all ARB prescriptions were included in the analysis.

^2^ Difference compared to valsartan.

Table S 2 Comparative interrupted time series analysis of data between September 2016 and June 2020: Change in monthly percentage of angiotensin II receptor blocker (ARB) prescriptions for valsartan vs other ARBs after July 2018 ^1^

| Comparator | Pre-intervention trend (p value) ^2^ | Post-intervention level change (p value) ^2^ | Post-intervention trend change (p value) ^2^ |
| --- | --- | --- | --- |
| Candesartan | -0.1778 (0.0897) | -13.76 (<0.0001) | -0.9108 (<0.0001) |
| Irbesartan | 0.0289 (0.6159) | -7.88 (<0.0001) | -0.4548 (<0.0001) |
| Losartan | 0.0283 (0.6059) | -7.77 (<0.0001) | -0.4902 (<0.0001) |
| Olmesartan | 0.0018 (0.9736) | -7.76 (<0.0001) | -0.4892 (<0.0001) |
| Telmisartan | 0.0353 (0.5254) | -7.77 (<0.0001) | -0.5158 (<0.0001) |

^1^ Only ARBs with a minimum proportion of 2% of all ARB prescriptions were included in the analysis.

^2^ Difference compared to valsartan.

Table S 3 Comparative interrupted time series analysis of data between January 2014 and June 2020: Change in quarterly percentage of incident angiotensin II receptor blocker (ARB) use for valsartan vs other ARBs after July 2018 ^1^

| Comparator | Pre-intervention trend (p value) ^2^ | Post-intervention level change (p value) ^2^ | Post-intervention trend change (p value) ^2^ |
| --- | --- | --- | --- |
| Candesartan | -0.8294 (0.0017) | -33.81 (<0.0001) | 1.07 (0.3297) |
| Irbesartan | 0.0473 (0.676) | -16.77 (<0.0001) | 0.3306 (0.5287) |
| Losartan | 0.0388 (0.7441) | -16.89 (<0.0001) | 0.2505 (0.6486) |
| Olmesartan | 0.171 (0.264) | -18.68 (<0.0001) | 0.2602 (0.7095) |
| Telmisartan | 0.0505 (0.6779) | -16.10 (<0.0001) | 0.1086 (0.8465) |

^1^ Only ARBs with a minimum proportion of 2% of all ARB prescriptions were included in the analysis.

^2^ Difference compared to valsartan.

Table S 4 Comparative interrupted time series analysis of data between January 2017 and June 2020: Change in quarterly percentage of incident angiotensin II receptor blocker (ARB) use for valsartan vs other ARBs after July 2018 ^1^

| Comparator | Pre-intervention trend (p value) ^2^ | Post-intervention level change (p value) ^2^ | Post-intervention trend change (p value) ^2^ |
| --- | --- | --- | --- |
| Candesartan | -3.11 (0.0111) | -25.79 (0.0012) | 3.35 (0.0394) |
| Irbesartan | -1.38 (0.0288) | -12.44 (0.0025) | 1.76 (0.0443) |
| Losartan | -1.55 (0.0142) | -11.98 (0.0025) | 1.84 (0.0321) |
| Olmesartan | -1.82 (0.0033) | -12.07 (0.0013) | 2.26 (0.0074) |
| Telmisartan | -1.59 (0.0135) | -11.51 (0.0036) | 1.74 (0.0421) |

^1^ Only ARBs with a minimum proportion of 2% of all ARB prescriptions were included in the analysis.

^2^ Difference compared to valsartan.
